# Clinical and genetic analysis of methylmalonic aciduria in 60 patients from Southern China: a single center retrospective study

**DOI:** 10.1186/s13023-025-03585-8

**Published:** 2025-02-07

**Authors:** Hiba Abid, Areeba Abid, Sarah Sohail, Sara Jawaid

**Affiliations:** Jinnah Sindh Medical University, Karachi City, V22W+F2H، Rafiqui H.J, Iqbal Shaheed Rd Karachi Cantonment, Karachi, 75510 Sindh Pakistan

**Keywords:** Methylmalonic aciduria, Treatment, Liver disease

## Abstract

We read with interest the recent publication on methylmalonic aciduria (MMA) and commend the authors for their outstanding contribution. This letter aims to further build upon their work by emphasizing additional aspects to enhance clinical relevance and diagnostic precision. We highlight the variability in serum MMA levels due to dietary intake, renal function, and external factors, advocating for the integration of supplementary diagnostic tools, such as the 1–13 C-propionate oxidation breath test, alongside biomarkers like fibroblast growth factor 21, growth differentiation factor 15, and lipocalin-2, to improve diagnostic accuracy. Additionally, we discuss the limitations of first-tier newborn screening tests due to high false positive rates and recommend second-tier testing using liquid chromatography coupled with tandem mass spectrometry to increase specificity and reduce false positives. Moreover, we address the underestimation of liver dysfunction in MMA patients, noting the need for longitudinal follow-up to capture the progression of liver function abnormalities. This critique is intended to constructively expand the authors’ findings and underscore the importance of a comprehensive diagnostic and management approach to MMA, ultimately improving patient outcomes.

**Supplementary Information:**

The online version contains supplementary material available at 10.1186/s13023-025-03585-8.

To the editor,

It was truly a delight to read this study by Ling Su et al. [1] as their impeccable efforts are clearly reflected in their work. Such a study requires immense energy and the ability to explore all relevant variables and precise details. We have extensively gone through the research [1], where this study deserves immense appreciation; we have come up with certain additional points that could have been included to validate this study. This is only to improvise and add to their work because as healthcare professionals it is our duty to strive for the betterment of human life.

This study [1] included all the necessary tests and analyses for confirming the diagnosis of patients with either combined or isolated methylmalonic aciduria. This shall furnish a comprehensive report. Nevertheless, one must consider that serum MMA levels may be very differently altered, depending on nutritional intake, exogenous supply, or renal function. Diagnostic tools such as gene panels and urine organic acid analysis remain critical for biochemical and genetic confirmation of MMA, but supplementary methods, such as the 1–13 C-propionate oxidation breath test, can provide non-invasive insights into mitochondrial dysfunction via biomarkers such as fibroblast growth factor 21 (FGF21), growth differentiation factor 15 (GDF15), and lipocalin-2. These integrated approaches may considerably enhance diagnostic accuracy and reduce false positives [2].

Secondly, the 6 asymptomatic cases of MMA were identified in newborns via newborn screening (NBS) [1]; however, this study [3] discusses the occurrence of a false positive in newborn. Since the levels of C3 are elevated and are not considered specific markers for these disorders, many false positives may occur. Although the introduction of analyte ratios, such as propionylcarnitine/acetylcarnitine (C3/C2) and C3/palmitoylcarnitine (C3/C16), has increased specificity, it still fails to completely resolve the diagnostic dilemma posed between propionic aciduria (PA) and MMAs, and a large number of false positives still exist. Therefore, it is strongly recommended to conduct secondary testing for all screen-positive cases to reduce the number of false positives in the first-order screening. Such second-tier tests, which are performed mainly with liquid chromatography coupled with tandem mass spectrometry, can also detect other analytes that are not assayed in the first line NBS test; because these are more specific markers for the suspicion of disease, they can be used to interpret abnormal results more effectively. The case described in the report culminated in vitamin B12 deficiency, and the patient’s neurological symptoms responded to cyanocobalamin treatment. This further stresses the need for confirmatory tests in neonates by secondary testing and considering external factors of partial enzyme deficiencies, maternal defects, nutritional deficiency, medication, and prematurity, so that timely and proper treatments may be provided [3].

Moreover, the authors also reported that liver function in severe MMA patients was ‘almost normal’ [1]. However, the retrospective nature of this study [1] may reduce the strength of the assessment of liver function because the evaluation of it would depend on when the tests were conducted and their frequency. Since liver dysfunction may occur in MMA and may not be immediately evident, increased longitudinal follow up would provide for a more sensitive determination of changes in liver function status over time [4]. Although clinical presentation was what the original article centered on, future studies with serial assessments of liver function might offer a more complete understanding of MMA’s progression and clinical course.

## Electronic supplementary material

Below is the link to the electronic supplementary material.

Supplementary Material 1

## Data Availability

All correspondence data are publicly available in databases such as PubMed.

## Abbreviations

MMA: Methylmalonic aciduria
POBT: 1–13 C-propionate oxidation breath test
FGF21: fibroblast growth factor 21
GDF15: growth differentiation factor 15
LCN2: lipocalin-2
NBS: Newborn screening
C3: propionylcarnitine
C2: acetyl carnitine
C16: palmitoyl carnitine
PA: propionic aciduria
LC-MS/MS: Liquid chromatography coupled to tandem mass spectrometry
