# Supplementary material for: Clinical and genetic analysis of methylmalonic aciduria in 60 patients from Southern China: a single center retrospective study
Source: Orphanet J Rare Dis. 2025 Feb 7;20:54. doi: 10.1186/s13023-025-03585-8 (PMC11804001; doi:10.1186/s13023-025-03585-8)
Supplement: Supplementary file 1 — Supplementary Material 1 [file 13023_2025_3585_MOESM1_ESM.docx]

**COVER LETTER**

Areeba Abid

Email: [areebaabid0101@gmail.com](mailto:areebaabid0101@gmail.com)

Institution: Jinnah Sindh Medical University

Address: V22W+F2H، Rafiqui H.J, Iqbal Shaheed Rd, Karachi Cantonment Karachi, Karachi City, Sindh 75510

Country: Pakistan

31-8-2024

To,

Editor-in-Chief

Orphanet Journal of Rare Diseases ,

Dear Editor,

I am pleased to submit our manuscript titled "Letter to the editor: Clinical and genetic analysis of methylmalonic aciduria in 60 patients from Southern China: a single center retrospective study" for consideration in the Orphanet Journal of Rare Diseases. We deeply appreciate the work of Ling Su et al. and aim to contribute further insights to enhance the understanding and clinical relevance of their study.

We confirm that this manuscript has not been previously submitted to or published in any other journal.

Thank you for your consideration of our submission. We look forward to your feedback.

Sincerely,

Areeba Abid,

Jinnah Sindh Medical University,

areebaabid0101@gmail.com.
